# Thought Leader Perspectives on the Benefits, Barriers, and Enablers for Routinely Collected Electronic Health Data to Support Professional Development: Qualitative Study

**DOI:** 10.2196/40685

**Published:** 2023-02-16

**Authors:** Bernard Bucalon, Emma Whitelock-Wainwright, Chris Williams, Jeanette Conley, Martin Veysey, Judy Kay, Tim Shaw

**Affiliations:** 1 Human Centred Technology Research Cluster School of Computer Science The University of Sydney Sydney Australia; 2 Centre for Learning Analytics Faculty of Information Technology Monash University Melbourne Australia; 3 Adventist HealthCare Limited Wahroonga Australia; 4 Division of Medicine Royal Darwin Hospital Tiwi Australia; 5 Research in Implementation Science and e-Health Group Faculty of Medicine and Health The University of Sydney Sydney Australia

**Keywords:** practice analytics, data visualization, continuing professional development, professional practice, reflective practice, lifelong learning, electronic health records, EHR

## Abstract

**Background:**

Hospitals routinely collect large amounts of administrative data such as length of stay, 28-day readmissions, and hospital-acquired complications; yet, these data are underused for continuing professional development (CPD). First, these clinical indicators are rarely reviewed outside of existing quality and safety reporting. Second, many medical specialists view their CPD requirements as time-consuming, having minimal impact on practice change and improving patient outcomes. There is an opportunity to build new user interfaces based on these data, designed to support individual and group reflection. Data-informed reflective practice has the potential to generate new insights about performance, bridging the gap between CPD and clinical practice.

**Objective:**

This study aims to understand why routinely collected administrative data have not yet become widely used to support reflective practice and lifelong learning.

**Methods:**

We conducted semistructured interviews (N=19) with thought leaders from a range of backgrounds, including clinicians, surgeons, chief medical officers, information and communications technology professionals, informaticians, researchers, and leaders from related industries. Interviews were thematically analyzed by 2 independent coders.

**Results:**

Respondents identified visibility of outcomes, peer comparison, group reflective discussions, and practice change as potential benefits. The key barriers included legacy technology, distrust with data quality, privacy, data misinterpretation, and team culture. Respondents suggested recruiting local champions for co-design, presenting data for understanding rather than information, coaching by specialty group leaders, and timely reflection linked to CPD as enablers to successful implementation.

**Conclusions:**

Overall, there was consensus among thought leaders, bringing together insights from diverse backgrounds and medical jurisdictions. We found that clinicians are interested in repurposing administrative data for professional development despite concerns with underlying data quality, privacy, legacy technology, and visual presentation. They prefer group reflection led by supportive specialty group leaders, rather than individual reflection. Our findings provide novel insights into the specific benefits, barriers, and benefits of potential reflective practice interfaces based on these data sets. They can inform the design of new models of in-hospital reflection linked to the annual CPD planning-recording-reflection cycle.

## Introduction

### Continuing Professional Development

There is growing recognition that routinely collected administrative information can be repurposed to provide the foundation for meaningful professional development that is grounded in rich data. Health practitioners are interested in reusing data such as length of stay, readmissions, and complications for personalized professional development [1]. At present, however, clinicians have relatively limited access to these data outside of external quality and safety reporting. Little is known on how the data can be used for collaborative learning [2,3]. There are several recognized system design challenges. First, reuse of administrative data for performance feedback introduces data quality issues, resulting in distrust of underlying clinical indicators [4]. Second, new health care information technology solutions require sustained support to ensure engagement and integration into practice [5]. Finally, clarity over data governance and access is required to address concerns that data would be used punitively rather than formatively for learning and development [6]. Improved access to previously fragmented and inaccessible data sets could provide new forms of evidence to meet annual professional development reporting requirements.

Continuing professional development (CPD) is well-recognized as critical for doctors to practice competently and ethically throughout their working lives. Practicing specialist clinicians need to undertake regular self-assessment to identify areas for improvement. Best practice CPD frameworks are based on adult learning principles of reflective practice [7] and lifelong learning [8]. Regular performance feedback, collaboration with peers, and self-reflection are among the cornerstones of lifelong learning [9]. Accordingly, medical regulators have shifted from traditional educational activities, that is, conferences, online courses, and workshops, to activities where clinicians review their performance and measure patient health outcomes [10-12]. This can be based on peer review, multisource feedback, and mentoring or coaching. Patient outcomes can be measured through audit and feedback (A&F) [13] and analyzed through morbidity and mortality (M&M) meetings or reflective practice.

Currently, doctors may find CPD becomes a “tick-the-box” exercise for compliance rather than for meaningful learning and behavioral change [14]. Clinicians can find it difficult to obtain evidence and record activities of participation, such as attendance at internal multidisciplinary team meetings. They feel CPD frameworks are complex, inflexible, and unrealistic [15,16]. Providing easy access to data about their performance presents an opportunity to address some of the barriers to high-quality CPD.

### Background and Significance

Our work aims to explore the potential for doctors to perform reflective CPD based on access to interfaces built on routinely collected administrative data. Although there is a recognition of its potential, there is a small but growing number of initiatives [17]. It is therefore timely to learn from these initiatives. As such, we designed a study to interview thought leaders internationally in addition to those in Australia to capture in-depth information about the experiences of authentic practice. The maintenance of professional competence varies internationally. In general, it involves the completion of educational and assessment activities related to reviewing clinician performance and patient outcomes. In the United States, CPD and continuing medical education activities (CME) include the use of knowledge testing for summative purposes, with a focus on underperformance, while other programs in the United Kingdom and Australia only use formative assessment and focus on development [18]. Despite these differences, investigating the benefits, barriers, and enablers to creating reflective practice tools is of international significance, as there is growing interest in how best to use evidence and health data to inform decision-making in health care delivery [19].

### Objective

Our study aims to gain understanding of the reasons that routinely collected administrative data have not yet become widely used to support reflective practice and lifelong learning. To do this, we explored the following research questions (RQs).

RQ1: What are the potential benefits of these reflective practice data tools?

RQ2: What are the barriers to the successful implementation?

RQ3: What are the enablers for such tools?

We interviewed thought leaders, asking them to reflect on their experiences and to identify the benefits of reflective practice interfaces based on administrative data. We then asked them to identify the barriers and enablers to implementation to capture the key considerations for their design.

## Methods

We used a qualitative methodology based on semistructured interviews. We designed the interview around the 3 RQs and asked background questions to capture data about their current role and professional and academic experience (Multimedia Appendix 1).

### Recruitment

We recruited a purposeful sample of thought leaders [20]. Table 1 shows the diversity of their job categories, specialty, and country. We sought thought leaders with experience in using, implementing, or evaluating static reports and interactive dashboards. We used 3 recruitment strategies. First, we invited authors cited in a recent scoping review [17] for their experiences in design, implementation, and evaluation. They are authors from diverse countries, representing different medical jurisdictions. The second recruitment group was from the research partners in the Practice Analytics program within the Digital Health Cooperative Research Centre (DHCRC). The DHCRC Practice Analytics program was established to explore the reuse of data from disparate hospital systems to support reflective practice, understand clinical variation, and improve patient health outcomes [21]. We recruited these participants from DHCRC for their relevant experience and their leadership roles within their organization. Research partners were based in Australia; therefore, their views provide depth in 1 country, complementing the first group. In total, 43 email invitations were sent to potential interviewees who met the inclusion criteria. Of the 43 invitations, 19 interviews were scheduled and conducted for the study. We did not receive a response from 22 of the email invitations. We had 2 more potential interviewees accept the invitation initially; one was unable to confirm a time for the interview and the other eventually declined to be part of the study.

Finally, we conducted snowball recruitment by asking each participant to recommend other suitable thought leaders. The sample size was determined by the diversity of the roles of the participants and when no new insights emerged from the interviews [22].

**Table 1:** Characteristics of the participants (N=19).

ID	Current job category	Current specialty	Clinical experience	Country
P01	Data analyst	Information and Communications Technology	No	Australia
P02	Technology administrator	Information and Communications Technology	No	Australia
P03	Chief Medical Officer	Medical administration	Yes	Australia
P04	Chief Medical Officer	Medical administration	Yes	Australia
P05	Chief Nursing Officer	Nursing administration	Yes	Australia
P06	Researcher	eHealth	No	Australia
P07	Researcher	Cancer	Yes	Canada
P08	Researcher	Clinical Decision-Making	No	United Kingdom
P09	Researcher	Learning Health Systems	No	Netherlands
P10	Researcher	Urology	Yes	United States
P11	Clinician/Researcher	Palliative Medicine, General and Acute Care Medicine	Yes	Australia
P12	Clinician	Urology	Yes	Australia
P13	Clinician	Gastroenterology, General and Acute Care Medicine	Yes	Australia/United Kingdom
P14	Clinician/Medical educator	Psychiatry	Yes	Australia
P15	Clinical informatician/Clinician	Informatics, Pediatrics	Yes	Australia
P16	Clinical informatician	Informatics	Yes	Australia
P17	Clinical informatician	Informatics	Yes	Australia
P18	Surgeon/Industry leader	Surgical Oncology	Yes	Canada
P19	Industry leader	Information and Communications Technology	Yes	Australia

### Data Collection

We used email to receive signed consent at the start of each interview and asked for verbal consent for audio recording. At the end of the interview, participants were invited to nominate other thought leaders for this study. The first author conducted the interviews online, and these ran for 45-60 minutes. The audio recordings were transcribed and then deidentified before analysis.

### Data Analysis

BB and EWW conducted unstructured thematic analysis on the interview transcripts to identify themes and subthemes [23]. The thematic analysis process involved 6 steps: (1) understanding the data by reading the transcripts and making notes, (2) generating an initial set of basic codes based on the RQs, (3) identifying top-level themes to categorize codes, (4) reorganizing themes and codes, (5) consolidating themes into their simplest form or idea, and (6) summarizing the findings. Both coders independently coded 1 transcript to generate an initial coding scheme. They then met to discuss their schemas and to resolve conflicts in coding. These steps were repeated 4 times until a consensus was reached. TS was the final arbiter where a transcript’s line code could not be resolved. Once the final coding scheme was agreed upon, the first author completed the coding for the remaining 15 interview transcripts. The coders met 3 times to organize the lower-level codes into coherent themes and subthemes. The 2 main coders then refined the final themes and subthemes structure to reduce redundancy and emphasize groupings. Finally, illustrative quotes were identified that best represented each theme.

### Ethics Approval

This study was granted ethics approval by The University of Sydney Human Research Ethics Committee (protocol/project: 2021/016). Care was taken during participant recruitment to ensure informed consent was obtained. BB distributed the ethics committee–approved email invitation to prospective participants. The coinvestigators ensured that the prospective participants contacted first met the inclusion criteria and that there were no existing conflicts of interest with the interviewer. Participants were able to ask clarifying questions about the study via email and before the interview recording commenced. Participant privacy and confidentiality were maintained by deidentifying the interview transcripts and audio recordings, replacing names with unique identifiers, for example, P01. Participants were not compensated for their involvement in the research study.

## Results

We present the results by highlighting the emergent themes from the interviews. For each RQ, we summarize the related themes identified. Although we identified clear themes and found little disagreement between job categories, there were differences between individual participant experiences. Illustrative quotes from the transcripts are provided for each of the themes (Multimedia Appendix 2).

### RQ1: What Are the Potential Benefits of Reflective Practice Data Tools?

The key benefit identified was that the access to new data sources improved the visibility of outcomes for all stakeholders. The visibility of outcomes allowed individual clinicians to compare their performance with peers or established benchmarks. The transparency of outcomes allowed specialty groups to identify cases of interest to support reflective discussions. Participants valued how the increased visibility of outcomes and group reflection led to practice change. Table 2 shows the illustrative quotes that identified potential benefits.

**Table 2:** Illustrative quotes on the potential benefits of the reflective practice data tools.

Theme	Quote
Peer comparison	…*I think it’s vitally important and it’s one of the things we try and do [...] and not only individuals, but also as groups or departments to highlight areas where there could be room for improvement. For example, we can look at length of stay for individual doctors versus the average. We can look at pressure areas, with falls. It’s really a very important and useful tool.* [P12, Clinician, Australia] …*The problem with sending data to individuals, it’s only useful if you send them the comparisons, because otherwise you might think you’re doing fine. But if you don’t know where you sit against your peers locally and your peers nationally, you don’t know how it is.* [P13, Clinician, Australia/United Kingdom]
Reflective group discussions	…*Things like length of stay, hospital acquired complications, and 28-day readmissions generate sufficient patients of interest to make a very robust discussion. So those 3 indicators really don’t say much about the outcome for the specialist on their own. But they highlight patients that are worth discussing as a craft (specialty) group.* [P17, Clinical informatician, Australia]
Practice change	…*If I’m trying to drive some sort of change through a specialty, we might pick out some data of interest and then show them what the data is now, and then decide together on a project - that we might be working on some sort of quality improvement project. Then track the same data over time. [...] So that’s another way we use data.* [P03, Chief Medical Officer, Australia] …*I think what happens is that we see that data becomes a nice to have, not that data becomes the thing that drives the change or drives the practice. I think really that the pendulum is still swinging and we need it to swing to the stage where data is the key thing behind driving all things, the decisions that we make, whether that be financial, patient flow, even clinical.* [P15, Informatician/Clinician, Australia]

#### Peer Comparison

Examples of peer comparison included league tables and benchmarking outcomes locally or against external data sets. However, some highlighted that peer comparison and competitiveness could be damaging. They acknowledged there were benefits for peer comparison on a unit level but felt individual competitiveness “goes slightly against the grain” as “we should be working collaboratively in health care” to improve performance and outcomes [P08]. Another participant noted that there may be a risk of a clinician reviewing their own data in isolation. They explained that with comparative data, individuals may misinterpret their own data; so, there is value in clinicians reviewing their data with peers.

#### Reflective Group Discussions

There was agreement that access to deidentified individual and group performance data for benchmarking supported robust team reflective discussions. One informatician noted that the goal is to discuss interesting patient cases rather than singling out individual clinicians.

#### Practice Change

Most of the participants identified changes to practice as a key benefit of reviewing routinely collected hospital data. This sentiment was shared across the different job categories interviewed. They stressed that having access to data, visibility of outcomes, and robust reflective discussions on interesting cases can lead to changes in practice that ultimately improve patient outcomes. One clinician stressed that while it is now easier to provide data for practitioners to review their performance, translating data insights to drive practice change is still a complex process. Other participants focused on the technology benefits of a new functionality to support the exploration of data, such as having the ability to drill down to case level information, quick searching, and filtering of large amounts of patient episode data, which previously required manual reporting by a data analyst or informatician. Finally, participants remarked on the efficiency improvements gained through new dashboard interfaces and electronic reports that saved time and reduced costs.

### RQ2: What are the Barriers to Successful Implementation?

The key barriers identified were both technical and human-centered. Participants had concerns with the existing technology and the quality of underlying data. They reflected on their experiences with reports and dashboards and were critical of the design of the data presented. Participants highlighted team culture as a barrier to conducting robust group reflective discussions. Table 3 shows the illustrative quotes related to the barriers to implementation.

**Table 3:** Illustrative quotes on the barriers to successful implementation.

Theme	Quote
Legacy technology and fragmented systems	…*So that’s another technical challenge and then there’s challenges with getting the data out of the medical records. I’m sure you’ve run into this too. If you’ve worked, actually worked with hospitals, but that’s a process by itself.* [P10, Researcher, United States]
Data quality, privacy, and lack of trust with underlying data	…*They’d see deidentified data of their colleagues. So it was the surgeon and their chief of staff [who] would also get the data. Then the idea is, would someone act on it? Especially when things are administrative claims type of data, you get a lot of questions about the quality of the data and that becomes a big barrier. So if people don’t trust the data, they’re going to ignore it.* [P07, Researcher, Canada] …*There’s then working with the specialists particularly the craft (specialty) group leaders to make sure that they’re comfortable with the process. They need to know what data will be presented, and [have] confidence that those indicators are reasonable and valid. They need to know what the consequence of having the data shared is.* [P13, Clinician, Australia/United Kingdom] …*I am wholeheartedly in support of it. We actually put this into practice and published a paper back in 2014 on this issue. It’s a difficult process, it’s difficult to get engagement. It’s difficult to maintain confidentiality. It’s not difficult, but you have to be very careful about maintaining patient confidentiality. So I’m supportive of it, but it’s not an easy task. It’s not something that’s a slam dunk, as we would say.* [P18, Surgeon/Industry leader, Canada]
Presentation and (mis)interpretation	…*Because it’s really easy to have and to look at the same data with different systems and set up your visualizations slightly differently to give people different impressions or to create confusion in what’s right. When you create that confusion, you bring in again more detractors who claim the data is wrong rather than actually the way the system is being applied, it’s not being then used logically.* [P12, Clinician, Australia]
Team culture and openness to group reflective discussion	…*The issues around data for improvement rather than judgment are real. Even though clinical teams want to improve, there’s always going to be a chance that you disintegrate into a culture of blaming people or being critical of people based on, often small, data sets or small numbers of stuff rather than looking at, and that can be a powerful problem*. [P08, Researcher, United Kingdom]

#### Legacy Technology and Fragmented Systems

Aging technology infrastructure and fragmented data sets within hospitals were identified as an important barrier. The incompatibility and complexity of the systems make it difficult for informaticians and clinicians to drill down to review individual cases. They also limit hospitals’ ability to implement new solutions. Some staff clinicians do not have access to a searchable database within their practice site, and another participant stressed the difficulty of extracting data from electronic medical records for individual and group reflection.

#### Data Quality, Privacy, and Lack of Trust With Underlying Data

Particular emphasis was put on the distrust with the underlying administrative data generated from day-to-day operations, such as length of stay, readmissions, and complications. However, some participants felt that the concerns around data quality were unfounded. They highlighted that the quality of the data is largely dependent on the documentation of medical records, which is later coded into the system. Interestingly, 3 participants suggested that the average practitioner may not be aware that their poor documentation may introduce coding errors, which ultimately impacts the overall quality of the data. Further, even if the data met the quality standards of clinicians, use in team meetings would likely depend on the openness of specialty group leaders.

Nine participants conveyed their concerns on doctor privacy and confidentiality. Although they were supportive of repurposing administrative data for reflection and professional development, both clinical and nonclinical participants highlighted the need for the performance data about team members to be deidentified. One informatician/pediatrician noted that smaller teams would increase the risk of identifying the consulting physician based on the rare conditions and specific medications for the patient episode.

#### Presentation and (Mis)Interpretation

Static reports and interactive dashboards were either overloaded with data or were too complex, leading to misinterpretation. One clinician remarked how the confusion created from the poor presentation may contribute to the concerns regarding data quality. The difficulties with interpretation were compounded by the varying levels of data literacy. Participants noted that designers should be mindful of data presented on reports, visualizations, and dashboards to ensure comprehension and minimize confusion. The problem of individual clinician attribution as part of a wider care team was also identified as a barrier. This occurs when multiple clinicians from either the same or different specialties, as part of a multidisciplinary team, were involved in a single patient episode of care.

#### Team Culture and Openness to Group Reflective Discussions

Establishing the right team culture was integral to promoting robust group reflective discussions. Participants felt that a punitive approach to reviewing performance data was not conducive to peer sharing and learning. Ultimately, the success of any performance data tool is underpinned by the culture of the teams supporting its use. Participants acknowledged that the varying levels of individuals’ engagement with their own data can be overcome by proactive specialty group leaders. For team members who may be hesitant to share their data with peers, these group leaders are crucial in coaching them to be more open to contributing to group reflective discussions. Unsurprisingly, participants also stressed that clinicians are too busy to be involved with the design and evaluation, resulting in new tools deployed with minimal end user feedback. The lack of adequate infrastructure and support staff to assist the rollout of new systems was also mentioned. Thought leaders also conveyed the link between these issues and concerns around the privacy and confidentiality of practitioners, suggesting that practitioners are hesitant to engage with new tools where the data access rules within the hospital were unclear.

### RQ3: What Are the Enablers to Such Tools?

The key enablers that surfaced were human-centered. Participants recommended identifying local champions to engage clinicians throughout the design process. They noted the important role of designers of new interfaces to prioritize clinicians’ understanding rather than simply sharing data. Team culture was a focus of discussion in the interviews, as they felt it played an integral role in supporting new reflective practice tools. Table 4 shows the illustrative quotes related to the enablers to implementation.

**Table 4:** Illustrative quotes on the enablers to such tools.

Theme	Quote
Champions and co-design	…*Before anything is delivered, the key stakeholders are consulted to get their opinion in terms of what would be useful to them or if there was a suggestion of something to get their feedback. So they actually feel as if they also have some ownership in it.* [P05, Chief Nursing Officer, Australia]
Present data for understanding, not for information	…*Simpler is better. What you need to do is give them the information they need to be able to make the decision or make a change in practice, but not overload them. [...] I think what you do is you take the data and you give it back to the team in the way in which they’ve asked for it. Then you leave them to figure out how they act on the results of that because each team will operate slightly differently.* [P08, Researcher, United Kingdom] …*And it does get reported in finance reports I can assure you of that. But for us, if we’re reporting length of stay, it’ll be done at a clinician level and will be tied very much to the approach we’re taking, tying it to individual patient risk adjustment. So we’re using that HIPAA model of patient risk adjustment, and we’re going to drive our reporting at clinician level based on that.* [P13, Clinician, Australia/United Kingdom]
Coaching and facilitation	…*But if the goal is primarily to get the data out there to learn from it, I think it’s very possible to learn from imperfect data. But again, that requires a certain level of trust and it requires an environment in which people aren’t judged for quote unquote poor performance. But in which people are understanding of the fact that different people work in different settings with different patients in different situations.* [P09, Researcher, Netherlands] …*It is tough, you know, and it is educational. The process we go through is deliberately not pointing fingers at anyone. It’s saying: “You look different to your peers. Would you like to find out why?” It’s not saying “Oh my God, you’ve got a problem here.” Because we wanted to say to people, actually, “You should get used to looking at your own performance and simply saying you’re within the gray zone of the shaded zone of the funnel plot, means you’re okay.”* [P11, Clinician/Researcher, Australia]
Timely recording of reflections for continuing professional development	…*One of the problems, like today, I did 3 meetings where I could claim CPD. But that would mean going out of the system, going into another system, finding my login, working out which of the five groups or whatever it is in and I’m going to put it down as. Then by the time I’ve done that, I’ll say “bugger it” and just do it like I normally do it - in a year or so. I go to two meetings a week. I think to make it meaningful and useful that some form of.. if it could be done with that kind of reflections automatically reflected because I think that would be an encouragement for people to do it.* [P12, Clinician, Australia]

#### Champions and Co-Design

Participants suggested identifying local champions or key opinion leaders to assist with promoting wider engagement with end users. Interviewees did acknowledge the difficulty of recruiting clinicians for consultation interviews and focus groups due to being time poor. Champions and opinion leaders do not necessarily have to be heads of departments; they can be unofficial leaders—any staff member who is well-respected within a group. Practitioner input into all stages of the design and implementation was seen as an important success factor. They highlighted the need for involving end users throughout the process to ensure they take ownership of the tool and integrate the tool into their practice. Co-design could include clinicians identifying relevant clinical indicators and suggesting effective ways to present the data. Clinicians could provide feedback on the reports and dashboards in development, thereby reducing errors and unanticipated consequences.

#### Present Data for Understanding, Not for Information

A third of the participants stressed that the presentation of the data should be optimized for understanding to minimize confusion. One respondent recommended minimal processing of the data, opting to provide the data as close to its original form back to practitioners. Therefore, specialty groups may transform and visualize the data specifically to their needs. Conversely, participants also stressed the importance of risk-adjusted clinical indicators to account for patient age and comorbidity or other factors that influence the results. When presenting comparative data, clinicians want to understand how CIs have been statistically weighted to take into account these factors so that teams are comparing “like for like.” Another participant emphasized the need for granularity of data. They wanted the ability to drill down and see the data broken down to be able to make sense of the data. Interestingly, a clinical informatician noted that some specialty group leaders prefer simple tools they are familiar with, such as Excel spreadsheets and PowerPoint slides, suggesting that access to data could be improved simply by using existing familiar tools.

#### Coaching and Facilitation to Support the Technology

Although participants saw value in individual specialists reviewing their own performance, they preferred having regular structured reflective discussions to embed the tools into practice. Respondents reflected on their experiences with tools, and data “dropped” onto them without any formal structures such as coaching or support to implement the change. One clinician described their experience where the team culture was not based on blame but rather sharing and learning.

#### Timely Recording of Reflections for CPD

Some respondents suggested that the reflection on practice undertaken within hospitals could be better captured for recording CPD. Currently, the systems are separate, which make it cumbersome to log reflections directly after a meeting. They suggested that a streamlined process could make the reflection more meaningful. Other enablers that emerged related to leveraging competition between practitioners. A chief medical officer remarked that league tables can be used to build healthy competition between clinicians in specialty groups. A researcher noted the importance of having robust data governance within the hospital to support the proper access and sharing of performance data for reflective practice. Other enablers related to the adequate provision of information technology support to specialty group leaders and individual clinicians during implementation.

## Discussion

This qualitative study captured the perspectives of Australian and international thought leaders on the potential benefits, barriers, and enablers of repurposing routinely collected administrative data sets for reflective practice interfaces.

### Proposed Benefits

Reflective practice interfaces are used in a diverse range of specialty domains, though they lack the best practice guidelines for a consistent end user experience. Thought leaders suggested enhanced visibility of outcomes, peer comparison, reflective discussion, and practice change as the main benefits. Transparency in the outcomes and peer comparisons play an important role in helping clinicians identify discrepancies between current and desirable practice and improve self-assessments [24]. Comparative data allow individual clinicians to reflect critically on their performance to gain new insights about their practice. In group settings, they can discuss patient cases of interest through peer learning. Regular reflection on data is central to lifelong learning that underpins the maintenance of many professional competence frameworks governing specialist registration [8,25,26]. Prior evaluations on the impact of clinical dashboards have shown some evidence of changing practice [27]. These dashboard interventions are largely based on electronic medical record and clinical registry data sets. Although our study focused on administrative data, we found thought leaders also view administrative data sets as rich sources to drive practice change [1]. Respondents were overall in agreement with the potential benefits of reflective practice interfaces based on administrative data. Our findings did reinforce the known barriers related to legacy technology infrastructure, siloed data sources, complex data visualizations, and cognitive overload [28].

### Team Culture and Group Reflection

Overcoming local cultural and organizational barriers to using reflective practice tools was viewed as important as the technical barriers to data presentation and technology infrastructure. A major theme was the culture and organizational structures required to implement and support the adoption of new reflective practice tools. Our findings are consistent with those in an earlier work on sustainable learning health systems, where transparent governance and clarity on the purpose of the data are considered vital for continued success [6]. Group leaders should reassure team members that the data will be used in formative learning contexts to support professional development rather than in a punitive manner, that is, singling out underperforming practitioners [1]. Only when team members feel safe, they will be willing to take interpersonal risks, be vulnerable, and give feedback to colleagues without being afraid of any negative consequences [25]. Respondents highlighted the difficulty of attributing individual performance to clinicians who work in highly collaborative specialties and multidisciplinary teams [29]. Despite this concern, there was a preference for group reflection over individual reflection. First, individual reflection is difficult, as practitioners may misinterpret the data or may lack the self-assessment skills required to identify areas for development. Second, robust discussions on patient cases of interest, supported by reflective practice tools, can be integrated into existing team meetings rather than adding work to clinicians’ busy schedules. These views were in line with the literature on the benefits of group reflection on performance improvement and practice change [26,30]. Reflective discussions on individual or team practice can be facilitated in both one-on-one meetings between a practitioner and a peer or facilitator or in a group meeting led by a facilitator [25].

### New Models of Reflection Linked to the CPD Cycle

Unlocking access to administrative data sets and requirements to review individual performance data provides an opportunity to develop new models of in-hospital reflection linked to professional development. Many medical practitioners engage in their CPD program as a compliance task rather than for professional learning and development [16]. A theme identified was the opportunity to streamline in-hospital review of data and subsequent recording of reflective discussions to meet the requirements related to practice-based review of performance. Assuming that data quality and governance concerns were addressed, respondents were supportive of the creation of new reflective data tools based on routinely collected administrative data. Although there has been extensive research into dashboards based on existing local processes such as A&F and M&M meetings, there is a gap in the literature linking in-hospital reflective interfaces and impacts to CPD [2].

There are similarities and differences with this type of reflection and reflection as part of A&F and M&M meetings. The existing tools are reactive in nature, starting with questions about practice performance, and data are collected accordingly. They are largely driven by external quality and safety reporting and hospital-level key performance indicators. The insights from the tools are discrete snapshots of performance and are retrospectively recorded for annual CPD reporting. In contrast, reflective practice tools provide a longitudinal view to professional development. They start with routinely collected data that are reviewed proactively. Patient cases of interest are identified to support robust team discussions that may lead to actions for improvement. Finally, they are driven by the internal professional goals of a clinician, documented in their personal development plan.

### Limitations

The participants interviewed represented views from a diverse range of fields and medical jurisdictions. We ensured that the participants interviewed had highly relevant backgrounds but strong Australian representation. As such, care should be taken in generalizing findings to local jurisdictions. Although the coding scheme was generated by 2 independent reviewers and reviewed by the coauthors, the majority of the transcripts ultimately were coded by 1 reviewer. To mitigate the researcher bias, coauthors were consulted multiple times during the coding and analysis process to verify the coding themes, interpreted themes, and conclusions.

### Conclusions

Our findings provide novel insights into the benefits, barriers, and enablers to repurposing administrative data for professional development. First, we identified that the potential benefits are visibility of outcomes, peer comparison, reflective group discussions, and practice change. Second, the barriers identified included legacy technology, distrust with data quality, privacy, data misinterpretation, and team culture. Third, the enablers identified included local champions participating in co-design, presenting data for understanding, specialty group leader coaching, and reflection linked to CPD. Despite issues with the underlying clinical indicators, clinicians are interested in reusing routinely collected data for professional development. We found that there was a strong preference for group reflective discussions supported by specialty group leaders over individual reflection. The application of these findings offers the potential to develop new models of in-hospital reflection linked to the annual CPD planning-recording-reporting cycle.

## Appendix

Multimedia Appendix 1 Interview guide.

Multimedia Appendix 2 All illustrative quotes.

## Abbreviations

A&F: audit and feedback
CPD: continuing professional development
DHCRC: Digital Health Cooperative Research Centre
M&M: morbidity and mortality
RQ: research question

## Data Availability

The data sets generated and analyzed during this study are not publicly available due to the conditions of The University of Sydney Human Research Ethics Committee, but we have provided a sample of the illustrative quotes in this paper and in the multimedia appendices.

## References

[1] Shaw T, Janssen A, Crampton R, O'Leary F, Hoyle P, Jones A, Shetty A, Gunja N, Ritchie AG, Spallek H, Solman A, Kay J, Makeham MA, Harnett P (2019). Attitudes of health professionals to using routinely collected clinical data for performance feedback and personalised professional development. Med J Aust.

[2] Vachon B, Désorcy Bruno, Gaboury I, Camirand M, Rodrigue J, Quesnel L, Guimond C, Labelle M, Huynh A, Grimshaw J (2015). Combining administrative data feedback, reflection and action planning to engage primary care professionals in quality improvement: qualitative assessment of short term program outcomes. BMC Health Serv Res.

[3] Mazzali C, Duca P (2015). Use of administrative data in healthcare research. Intern Emerg Med.

[4] Razzaghi H, Greenberg J, Bailey LC (2022). Developing a systematic approach to assessing data quality in secondary use of clinical data based on intended use. Learn Health Syst.

[5] Cresswell K, Sheikh A (2013). Organizational issues in the implementation and adoption of health information technology innovations: an interpretative review. Int J Med Inform.

[6] Enticott J, Braaf S, Johnson A, Jones A, Teede HJ (2020). Leaders' perspectives on learning health systems: a qualitative study. BMC Health Serv Res.

[7] DunnGalvin A, Cooper JB, Shorten G, Blum RH (2019). Applied reflective practice in medicine and anaesthesiology. Br J Anaesth.

[8] Cole M (2006). Learning through reflective practice: a professional approach to effective continuing professional development among healthcare professionals. Research in Post-Compulsory Education.

[9] Professional performance framework. Medical Board of Australia.

[10] Narayanan A, Farmer EA, Greco MJ (2018). Multisource feedback as part of the Medical Board of Australia's Professional Performance Framework: outcomes from a preliminary study. BMC Med Educ.

[11] Jeong D, Presseau J, ElChamaa R, Naumann DN, Mascaro C, Luconi F, Smith KM, Kitto S (2018). Barriers and Facilitators to Self-Directed Learning in Continuing Professional Development for Physicians in Canada. Academic Medicine.

[12] Karas M, Sheen NJL, North RV, Ryan B, Bullock A (2020). Continuing professional development requirements for UK health professionals: a scoping review. BMJ Open.

[13] Ivers N, Jamtvedt G, Flottorp S, Young JM, Odgaard-Jensen J, French SD, O'Brien MA, Johansen M, Grimshaw J, Oxman AD (2012). Audit and feedback: effects on professional practice and healthcare outcomes. Cochrane Database Syst Rev.

[14] McGivern G, Ferlie E (2016). Playing tick-box games: Interrelating defences in professional appraisal. Human Relations.

[15] Hanlon H, Prihodova L, Hoey H, Russell T, Donegan D, O'Shaughnessy Ann (2021). Attitudes, Perceived Benefits, and Experiences of Engagement With Professional Competence Schemes for Doctors in Ireland: Findings From a National Survey. J Contin Educ Health Prof.

[16] Wiese A, Galvin E, Korotchikova I, Bennett D (2022). Doctors' attitudes to maintenance of professional competence: A scoping review. Med Educ.

[17] Bucalon B, Shaw T, Brown K, Kay J (2022). State-of-the-art Dashboards on Clinical Indicator Data to Support Reflection on Practice: Scoping Review. JMIR Med Inform.

[18] Archer J, Pitt R, Nunn S, Regan DBS (2015). The evidence and options for medical revalidation in the Australian context. Medical Board of Australia.

[19] Melder A, Robinson T, McLoughlin I, Iedema R, Teede H (2020). An overview of healthcare improvement: unpacking the complexity for clinicians and managers in a learning health system. Intern Med J.

[20] Palinkas LA, Horwitz SM, Green CA, Wisdom JP, Duan N, Hoagwood K (2015). Purposeful Sampling for Qualitative Data Collection and Analysis in Mixed Method Implementation Research. Adm Policy Ment Health.

[21] Using practice analytics to understand variation and support reflective practice. Digital Health Cooperative Research Centre.

[22] Vasileiou K, Barnett J, Thorpe S, Young T (2018). Characterising and justifying sample size sufficiency in interview-based studies: systematic analysis of qualitative health research over a 15-year period. BMC Med Res Methodol.

[23] Braun V, Clarke V, Hayfield N, Terry G, Liamputtong P (2019). Thematic analysis. Handbook of Research Methods in Health Social Sciences.

[24] Gude WT, Roos-Blom M, van der Veer SN, Dongelmans DA, de Jonge E, Peek N, de Keizer NF (2019). Facilitating action planning within audit and feedback interventions: a mixed-methods process evaluation of an action implementation toolbox in intensive care. Implement Sci.

[25] Bindels E, Verberg C, Scherpbier A, Heeneman S, Lombarts K (2018). Reflection revisited: how physicians conceptualize and experience reflection in professional practice - a qualitative study. BMC Med Educ.

[26] Mann K, Gordon J, MacLeod A (2009). Reflection and reflective practice in health professions education: a systematic review. Adv Health Sci Educ Theory Pract.

[27] Tuti T, Nzinga J, Njoroge M, Brown B, Peek N, English M, Paton C, van der Veer SN (2017). A systematic review of electronic audit and feedback: intervention effectiveness and use of behaviour change theory. Implement Sci.

[28] Khairat SS, Dukkipati A, Lauria HA, Bice T, Travers D, Carson SS (2018). The Impact of Visualization Dashboards on Quality of Care and Clinician Satisfaction: Integrative Literature Review. JMIR Hum Factors.

[29] Sebok-Syer Stefanie S, Shaw JM, Asghar F, Panza M, Syer MD, Lingard L (2021). A scoping review of approaches for measuring 'interdependent' collaborative performances. Med Educ.

[30] Sandars J (2009). The use of reflection in medical education: AMEE Guide No. 44. Med Teach.

