# Supplementary material for: Thought Leader Perspectives on the Benefits, Barriers, and Enablers for Routinely Collected Electronic Health Data to Support Professional Development: Qualitative Study
Source: J Med Internet Res. 2023 Feb 16;25:e40685. doi: 10.2196/40685 (PMC9982719; doi:10.2196/40685)
Supplement: Multimedia Appendix 1 [file jmir_v25i1e40685_app1.docx]

**Multimedia Appendix 1- Interview guide**

| **Key question** | **Question prompts** |
| --- | --- |
| 1. What is your role and how long have you been in the role? | - What does your role entail? - How long have you been working in your field? - Can you tell me about your professional and/or academic background? |
| 1. What are your thoughts on individual clinicians and teams using routinely collected hospital data (quality/performance indicators) for performance improvement? | - What are the benefits? - What are the risks? - Can you name specific clinical indicators that clinicians have used for professional learning? |
| 1. Are you aware of any data tools (e.g. reports, dashboards, visualizations) used at your site to support individual or team performance improvement? | - What does the data tool mean? - How did the data tool come about? - Can you tell me about its use? - Can you tell me about experiences that give insight into how people can use the data tool? |
| 1. In an ideal world, clinical indicator data should support clinicians to gain insights about their own practice.  If you had a single magic wand, what clinical indicators would be on a dashboard to support the professional learning of clinicians? | - For individuals? - For teams/MDTs? - What features would the dashboard have? - How often would it be used? |
| 1. What do you think are the key barriers to the successful implementation of new data tools into practice? | - Any organizational barriers? - Any cultural barriers? - Any technical barriers? - Any regulatory barriers? |
| 1. What are some strategies you believe would support the successful implementation of a new data tool into practice? | - What about the people (stakeholders)? - What about the processes? - What about the technology? |
| 1. Do you have any questions or comments related to anything we discussed today? | - Would you like to recommend a colleague or contact you have that may be interested in participating in this study? |
