# Supplementary material for: Thought Leader Perspectives on the Benefits, Barriers, and Enablers for Routinely Collected Electronic Health Data to Support Professional Development: Qualitative Study
Source: J Med Internet Res. 2023 Feb 16;25:e40685. doi: 10.2196/40685 (PMC9982719; doi:10.2196/40685)
Supplement: Multimedia Appendix 2 [file jmir_v25i1e40685_app2.docx]

**Multimedia Appendix 2 - All illustrative quotes**

| **RQ** | **Theme** | **Quote** |
| --- | --- | --- |
| RQ1 | Peer comparison | “*So if you can show them data where it's either in a league table or somehow comparing between them and their colleagues, and maybe not just locally at this hospital, but state-wide or nationally or internationally. If they can see how they fit into that, that can be quite powerful data. It's powerful if they're doing well, but it's also powerful if they're not doing well.”* (P03-Chief Medical Officer) |
|  |  | *“I think that peer comparison, that competitiveness - again, this is my own personal view - I think there is benefit in comparing the performance of units and teams and people who look after a certain type of patient across hospitals. So I want to know if my unit, my critical care unit in London is performing as well as the critical care unit in Manchester. I do know that in certain areas they do. Historically, I think in the UK, we had a database which compared the performance of individual surgeons which I think can have some benefits to them. But personally, I think that's quite damaging. I actually personally think that peer to peer comparison and that sort of competitiveness goes slightly against the grain because what we should be doing in health care is working collaboratively to improve performance.”* (P08-Researcher-UK) |
|  |  | ***“****I think it's vitally important and it's one of the things we try and do [...] and not only individuals, but also as groups or departments to highlight areas where there could be room for improvement. For example we can look at length of stay for individual doctors versus the average. We can look at pressure areas, with falls. It's really a very important and useful tool.”* (P12-Clinician) |
|  |  | *“The problem with sending data to individuals, it's only useful if you send them the comparisons, because otherwise you might think you're doing fine. But if you don't know where you sit against your peers locally and your peers nationally, you don't know how it is.”* (P13-Clinician-AU/UK) |
|  | Reflective group discussions | *“Yes, data has its flaws, but data when used the right way can be beneficial, and very reflective. And so that change management piece is really important. As with all change management projects, they will always be a curve. There's some people who are way ahead of the game. And there's some people who lag behind in terms of adoption of data and understanding of that. So that's going to be a key role.”* (P15-Informatician/Clinician) |
|  |  | ***“****Things like length of stay, hospital acquired complications and 28-day readmissions generate sufficient patients of interest to make a very robust discussion. So those three indicators really don't say much about the outcome for the specialist on their own. But they highlight patients that are worth discussing as a craft (specialty) group.”* (P17-Clinical informatician) |
|  | Practice change | *“If I'm trying to drive some sort of change through a specialty, we might pick out some data of interest and then show them what the data is now, and then decide together on a project that we might be working on some sort of quality improvement project. Then track the same data over time. [...] So that's another way we use data.”* (P03-Chief Medical Officer) |
|  |  | *“I think what happens is that we see that data becomes a nice to have, not that data becomes the thing that drives the change or drives the practice. I think really that the pendulum is still swinging and we need it to swing to the stage where data is the key thing behind driving all things, the decisions that we make, whether that be financial, patient flow, even clinical.”* (P15-Informatician/Clinician) |
| RQ2 | Legacy technology and fragmented systems | *“Cost is probably one though. It's not something I encounter that often and I suspect it's probably the least, least biggest hurdle in some ways. Because I think there's a lot of off the shelf tools that can potentially be used to build dashboards that aren't that expensive.”* (P07-Researcher-CAN) |
|  |  | *“So that's another technical challenge and then there’s challenges with getting the data out of the medical records. I'm sure you've run into this too. If you've worked, actually worked with hospitals, but that's a process by itself.”* (P10-Researcher-US) |
|  | Data quality, privacy, and lack of trust with underlying data | *“They'd see de-identified data of their colleagues. So it was the surgeon and their chief of staff [who] would also get the data. Then the idea is, would someone act on it? Especially when things are administrative claims type of data, you get a lot of questions about the quality of the data and that becomes a big barrier. So if people don't trust the data, they're going to ignore it.” (P07-Researcher-CAN)* |
|  |  | *“There's then working with the specialists particularly the craft (specialty) group leaders to make sure that they're comfortable with the process. They need to know what data will be presented. Confidence that those indicators are reasonable and valid. They need to know what the consequence of having the data shared is.”* (P13-Clinician-AU/UK) |
|  |  | “*Often when we start looking at data (and [it] applies to as much as the administrative datasets), the first response of clinicians when they particularly see their outcomes are not the same or better than their peers; that the data is wrong. And my response to them is always ‘this is your data’. Some of the benefits of this approach is that allows people to go and actually review their data that they put into these systems.”* (P16-Informatician) |
|  |  | “I am wholeheartedly in support of it. We actually put this into practice and published a paper back in 2014 on this issue. It's a difficult process, it's difficult to get engagement. It's difficult to maintain confidentiality. It's not difficult, but you have to be very careful about maintaining patient confidentiality. So I'm supportive of it, but it's not an easy task. It's not something that's a slam dunk, as we would say.” (P18-Surgeon/Industry leader-CAN) |
|  | Presentation and (mis)interpretation | *“It's an education process and the culture isn't there. A key issue is the information literacy of the average clinician is actually really poor. And I say that because we put reports in front of people and listen to their interpretation of those reports that have been worked through with clinicians in order to generate them in the first place.''* (P06-Researcher) |
|  |  | *“So specifically related to what you said, reusing data. I think quality control and quality assurance of the data is a real challenge. The way that for example, people get attributed to a certain patient case is messy and really patients are cared for by a team. So to attribute certain data to an individual physician or any other healthcare provider is really challenging.”* (P10-Researcher) |
|  |  | *“Because it's really easy to have and to look at the same data with different systems and set up your visualizations slightly differently to give people different impressions or to create confusion in what's right. When you create that confusion, you bring in again more detractors who claim the data is wrong rather than actually the way the system is being applied, it’s not being then used logically.” (P12-Clinician)* |
|  | Team culture and openness to group reflective discussions | *“Yeah, but I think probably buy-in is more of a challenge than the technology, like it's just, I don't think hospitals are going to adopt these things unless they really feel their health workforce is demanding it or want it. And to that, I think we really need to figure out why the health workforce wants to use these tools beyond research, or you can make a really great research dashboard, but that's custom [to their needs].”* (P07-Researcher-CAN) |
|  |  | *“The issues around data for improvement rather than judgment are real. Even though clinical teams want to improve, there's always going to be a chance that you disintegrate into a culture of blaming people or being critical of people based on, often small, datasets or small numbers of stuff rather than looking at, and that can be a powerful problem.”* (P08-Researcher-UK) |
|  |  | *“Then there's the barrier about open disclosure or open discussion. Some specialists are very reticent to talk about complications that they've had even with their peers. So our experience has been that in preparation for the craft (specialty) group meetings, the craft (specialty) group leader often has to coach the individual specialist, particularly if the patient died or was a significant complication that the specialist is now remorseful about.”* (P17-Informatician) |
| RQ3 | Champions and co-design | *“Before anything is delivered, the key stakeholders are consulted to get their opinion in terms of what would be useful to them or if there was a suggestion of something to get their feedback. So they actually feel as if they also have some ownership in it.”* (P05-Chief Nursing Officer) |
|  |  | *“The other thing, this is still kind of leadership, but at the other sort of lower level, I think it really helps if you're implementing it in a team or teams or a department that the senior medical officer is really passionate and really like encouraging people to use these tools.”* (P06-Researcher) |
|  |  | *“It probably is the element of sufficient time that has to be given to the parties that are involved in it. So they've got an explanation and they have an understanding as to why it's required and they actually buy into the fact that it's important. Otherwise, it would just lead to… the human function in terms of it not really being believed in.”* (P07-Researcher-CAN) |
|  |  | *“And a lot of work on co-design. And I think that's another thing that people don't concentrate a lot on is that we have Tableau. So we put stuff together in Tableau and we give it to practitioners and then we're really surprised cause they don't use it. And that's because we haven't even bothered to ask them what information they need to do their job more effectively. And how could we give them that information in a way, which is helpful to them in their practice and how could we design it with them so that it really helps them in their practice.”* (P08-Researcher-UK) |
|  |  | *“I think it's even deeper though. I think it's important to engage your clinicians in those decisions, which metrics do you want to show to get their input. For example, we have some very useful insights about clinicians where I said it's important to also look at this countermeasure because otherwise you might get some kind of unanticipated consequence for people and start to discharge patients soon. So when they’re readmitted or there'll be higher mortality rates or so for these kinds of things.”* (P09-Researcher-NL) |
|  |  | *“So I think key opinion leaders, absolutely. Listening, listening and really walking people through so that they become information literate and making it clear that no question is too silly, too dumb but really working with people so that they are empowered to understand and then utilise this information.”* (P11-Clinician/Researcher) |
|  |  | *“You need either official or unofficial leaders and opinion leaders. Now that could be the chief of surgery, but there's lots of Chief of Surgery who are disrespected by the members of their department. So it has to be someone who has some gravitas with the group who is actually a champion for the department.”* (P18-Surgeon/Industry leader-CAN) |
|  | Present data for understanding, not for information | *“So you might have a broad figure, but it needs to be broken down in terms of actually getting a better picture of what it actually means. Then also a breakdown. So the number of falls, it could be that someone was lowered to the ground, which is completely different to somebody that's had a fall and fractured their hip. So you might have a broad figure, but it needs to be broken down in terms of actually getting a better picture of what it actually means.”* (P05-Chief Nursing Officer) |
|  |  | *“Simpler is better. What you need to do is give them the information they need to be able to make the decision or make a change in practice, but not overload them. [...] I think what you do is you take the data and you give it back to the team in the way in which they've asked for it. Then you leave them to figure out how they act on the results of that because each team will operate slightly differently.”* (P08-Researcher-UK) |
|  |  | *“Look most clinicians still fly blind and so anything is going to be better than nothing, as long as it's adjusted and you're comparing like for like.”* (P11-Clinician/Researcher) |
|  |  | *“And it does get reported in finance reports I can assure you of that. But for us, if we’re reporting length of stay, it'll be done at a clinician level and will be tied very much to the approach we’re taking is tying it to individual patient risk adjustment. So we're using that HIPAA model of patient risk adjustment, and we're going to drive our reporting at clinician level based on that”.* (P13-Clinician-AU/UK) |
|  |  | *“The other - I should've mentioned in when you talk about risks - often in data visualization, I think people try to make it too complicated. I have a view that a very simple dashboard with a simple amount of data on it is a lot more useful and gets a lot more improvement. Then a complex dashboard with 47 different types of graphs and data sources all mashed into a page, which is technically very satisfying for the person who's cleverly created that is not so useful in actually driving change.”* (P16-Clinical informatician) |
|  | Coaching and facilitation to support the technology | *“And then also people can have very good intentions. But it's not just the dropping of data. It's actually having some really defined touch points to actually be measuring and reporting back on improvements or where things have to be improved. Otherwise it can be felt that it is just data and nothing more than that. It's a cultural change program really that's required.''* (P05-Chief Nursing Officer) |
|  |  | *“But if the goal is primarily to get the data out there to learn from it, I think it's very possible to learn from imperfect data. But again, that requires a certain level of trust and it requires an environment in which people aren’t judged for quote unquote poor performance. But in which people are understanding of the fact that different people work in different settings with different patients in different situations.”* (P09-Researcher) |
|  |  | *“It is tough you know, and it is educational. The process we go through is deliberately not pointing fingers at anyone. It's saying: ‘You look different to your peers. Would you like to find out why?' It's not saying ‘Oh my God, you've got a problem here'. Because we wanted to say to people, actually, ‘You should get used to looking at your own performance and simply saying you're within the gray zone of the shaded zone of the funnel plot, means you're okay.’”* (P11-Clinician/Researcher) |
|  | Timely recording of reflection for CPD | ***“****One of the problems, like today, I did three meetings where I could claim CPD. But that would mean going out of the system, going into another system, finding my login, working out which of the five groups or whatever it is in and I’m going to put it down as. Then by the time I’ve done that, I’ll say ‘bugger it’ and just do it like I normally do it - in a year or so. I go to two meetings a week. I think to make it meaningful and useful that some form of.. if it could be done with that kind of reflections automatically reflected because I think that would be an encouragement for people to do it.”* (P12-Clinician) |
|  |  | *“It’s why I talked about at the beginning about appraisal and job planning, because it needs to be linked into that process. It probably should be linked. This is my interest, particularly in CPD and becoming important. It probably should be linked into remuneration. Because it should probably be that you can't continue to bill Medicare for items, unless you engage in a process to demonstrate you've engaged in that process.”* (P13-Clinician-AU/UK) |
|  |  | *“Craft (specialty) group meetings. A lot of them last two hours, so that's eight hours just from attendance. And then you've got a couple of hours preparing your case. For presentations. So you could easily find 20, 25 hours in reflective practice as part of your CPD.”* (P17-Clinical informatician) |
